# A Balanced Diet?: Selenium May Offset the Effects of Methylmercury on Cataract Development

**DOI:** 10.1289/ehp.118-a491b

**Published:** 2010-11

**Authors:** Naomi Lubick

**Affiliations:** **Naomi Lubick** is a freelance science writer based in Stockholm, Sweden, and Folsom, CA. She has written for *Environmental Science & Technology*, *Nature*, and *Earth*

Dietary exposure to mercury from fish has been posited as a risk factor for cataracts because some reports have suggested methylmercury accumulates in the lens of the eye. But selenium from other dietary sources may offset that damage, according to a study of communities in the Amazon basin **[***EHP* **118(11):1584–1589; Lemire et al.]**. The findings, while preliminary, hint at potential public health measures in areas where methylmercury-contaminated fish are a significant part of people’s diets.

With old age comes cataracts, particularly in latitudes like the Amazon, where higher ultraviolet radiation exposure and other environmental factors contribute to the clouding of the lenses in human eyes. And while surgical fixes exist for cataracts, people in isolated regions may not always have access to those options. Cataracts therefore are a major cause of blindness among older people in the Amazon.

The current study involved communities that eat fish from the Tapajós River, a tributary of the Amazon. People here have among the highest reported exposures to mercury in the world. Deforestation in the region leads to the release of natural inorganic mercury from soils into surface waters, where it is methylated and eventually ends up in fish.

Several hundred people voluntarily participated in the study, which entailed taking an overnight boat trip to a nearby city where participants gave blood samples and were examined by optometrists. In the end, 211 people over age 40 were included in the analysis. A third of them had age-related cataracts.

Low plasma selenium and high blood mercury each were associated with a higher prevalence of cataracts (over 2 and 4 times higher, respectively). The team calculated that the people with both low plasma selenium and high blood mercury were 16 times more likely to develop cataracts than the “optimum situation” group, which had both high plasma selenium and low blood mercury.

This is the first study known to associate high levels of methylmercury from fish consumption with increased occurrence of cataracts. The authors emphasize that other factors—such as differences in dietary intakes of antioxidants and vitamins—could confound their findings. Still, if the observed associations hold true in broader studies, public health interventions to alleviate cataracts in this Amazonian population must consider both the health benefits of fish consumption and the risks of the main source of dietary selenium in the region: brazil nuts, which also contain barium and strontium, heavy metals with their own hazards.

## Figures and Tables

**Figure:**
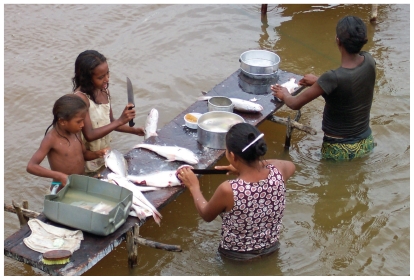
Women and children from a Tapajós village clean fish, a chief component of the local diet, which also includes rice, manioc flour, fruits, and brazil nuts.

